# Regulation of Insulin Resistance, Lipid Profile and Glucose Metabolism Associated with Polycystic Ovary Syndrome by *Tinospora cordifolia*

**DOI:** 10.3390/nu15102238

**Published:** 2023-05-09

**Authors:** Ritu Rani, Havagiray R. Chitme, Neha Kukreti, Pankaj Pant, Basel A. Abdel-Wahab, Masood Medleri Khateeb, Mohammed Shafiuddin Habeeb, Marwa B. Bakir

**Affiliations:** 1 Faculty of Pharmacy, DIT University, Dehradun 248009, Uttarakhand, India; 1000013859@dit.edu.in (R.R.); 1000013858@dit.edu.in (N.K.); pankaj.pant@dituniversity.edu.in (P.P.); 2 Department of Pharmacology, College of Pharmacy, Najran University, Najran P.O. Box 1988, Saudi Arabia; babdelnaem@nu.edu.sa (B.A.A.-W.); mmkhateeb@nu.edu.sa (M.M.K.); mshabeeb@nu.edu.sa (M.S.H.); 3 Department of Pharmacology, College of Medicine, Najran University, Najran P.O. Box 1988, Saudi Arabia; mbbakir@nu.edu.sa

**Keywords:** PCOS severity, insulin resistance, insulin sensitivity, guduchi satva, guduchi oil, guduchi hydroalcoholic extract

## Abstract

*Background:* The plant *Tinospora cordifolia* (TC), traditionally known as guduchi or giloy, is used for a number of health conditions as a nutritional supplement and rejuvenation medicine. Its nutritional supplementary products are traditionally recommended for a wide range of health issues, including diabetes, menstruation discomfort, fever, obesity, inflammation, and more. Unfortunately, there has not been extensive research into its effectiveness in treating or managing insulin resistance, lipid and carbohydrate metabolism, hormonal imbalance, and metabolic syndrome-associated polycystic ovary syndrome (PCOS). *Methods:* Consequently, the present study was designed to induce insulin resistance, dyslipidemia, hormonal abnormality, hyperglycemia, and menstrual disturbance of PCOS using dehydroepiandrosterone (DHEA) in mice and study the effect of oral TC extracts on these factors by using ancient and modern technologies. During the 21-day study, 6 mg/100 g/day of DHEA was given to female mice. Levels of glucose, insulin, lipids, and hormones were estimated. In addition to being seen with the naked eye, the morphological and microscopic changes were also observed on histology slides. *Results:* The study outcomes show that pretreatment with TC preparations significantly improved biochemical and histological abnormalities in female mice. Diestrus phase was only observed in DHEA-treated animals, while cornified epithelial cells were present in TC-treated mice. Pretreatment with TC satva showed significant (*p* < 0.001) reductions in body weight compared to placebo. Fasting blood glucose, 1-h OGTT, and 2-h OGTT levels were all significantly lower in TC satva- and oil-treated animals in comparison to the disease control group (*p* < 0.001). Treatment with TC extracts resulted in a normalization of estradiol, progesterone, and testosterone levels (*p* < 0.05). Treatment with TC extract improved lipid profiles (*p* < 0.001), LH/FSH ratios (*p* < 0.01), fasting insulin levels (*p* < 0.001), HOMA-IR (*p* < 0.001), HOMA-Beta (*p* < 0.001), and QUICKI (*p* < 0.001). Both macroscopic and microscopic alterations were seen to be restored after TC extract treatment. After being treated with TC satva, oil, and hydroalcoholic extract, the severity of PCOS decreased by 54.86%. *Conclusions:* These findings lead us to the conclusion that TC extracts and satva as nutritional supplements are useful for treating PCOS and associated symptoms. It is recommended that additional research be conducted to determine the molecular mechanism of action of TC nutritional supplements on PCOS-related changes in metabolic profiles. We also recommend further clinical studies to explore the clinical efficacy and effectiveness of TC nutritional supplements in treating and/or managing PCOS.

## 1. Introduction

Polycystic ovary syndrome (PCOS) is a leading cause of infertility in women due to hormonal imbalance. It manifests as polycystic ovaries, elevated testosterone levels, and infertility [1]. It is estimated that between 5% and 10% of women aged 18–44 may be affected by PCOS [2]. There are many prominent risk factors for this condition, including hereditary and environmental components, according to epidemiological studies [3]. Menstrual problems, infertility, diabetes, elevated levels of masculinizing hormones, a hyperinsulinemic metabolic syndrome, and endometrial cancer are all clinical symptoms of PCOS [4]. Menstrual dysfunction, acne, hirsutism, and infrequent or absent ovulation are all symptoms of hyperandrogenism associated with PCOS [5].

Increased luteinizing hormone (LH) or elevated blood insulin levels are considered to be two prominent contributors to the cause of PCOS [6]. Patients with PCOS usually undergo extensive clinical treatment, which may involve both lifestyle modification and pharmaceutical interventions. Treatment options for polycystic ovarian syndrome are severely limited. The symptoms of PCOS can be managed with symptomatic treatments [7]. The primary and realistic objectives of therapy in PCOS are fertility restoration, insulin resistance (IR) improvement, acne and hirsutism management, menstrual cycle regularization, and endometrial hyperplasia prevention [8]. Hot flashes, arthritis, joint or muscle discomfort, irritability, mood swings, melancholy, and bloating are some of the psychological and physiological side effects of contemporary PCOS treatment that can range from mild to severe [9].

Medicines produced from natural sources may be an impressive substitute therapy with a familiar mechanism of action for the creation of a powerful, safe, and cost-effective treatment agent for this ailment [9]. However, so far, there is scant scientific basis for the claims that complementary and alternative therapies are both safe and effective. This necessitates testing some unconventional methods for preventing and treating PCOS, such as medicines [7]. To this day, *Tinospora cordifolia* (TC) is one of Ayurveda’s most popular medicinal plants [10]. In Ayurvedic and ethnic medicine, TC has several medical uses due to its lack of side effects/minimal side effects and wide range of beneficial effects, including for periods, allergies, arthritis, fever, leprosy, diabetes, stress, and malaria. The stem has a number of medical purposes, including as a diuretic, a thirst quencher, a source of iron and vitamins, a jaundice cure, and a bitter stomachic. There is evidence that using a plant stem extract can assist with skin disorders. When combined with other drugs, TC serves as an antidote for snakebite and scorpion stings [11]. Some of the other ailments that this plant aids in treating include asthma, cough, wounds, and pneumonia. It is also found to be effective against cancer and provides an improvement in the immune system, protection of nerve cells, resistance to diabetes, a reduction in cholesterol, and protection of the liver. Evidence suggests that TC can help diabetic foot ulcers heal faster and mitigate some of the side effects of both chemotherapy and radiation [10].

As an anti-inflammatory herb, TC is believed to be extremely effective against PCOS due to the fact that insulin dysregulation and ovarian cysts have a common root cause, i.e., chronic mild inflammation in the tissues. Reducing IR increases metabolism and stimulates all tissues naturally [12]. The important constituents of TC are alkaloids (tinosporine, magnoflorine, berberine (BER), choline, jatrorrhizine, palmatine (PAL), tembeterine), flavonoids (quercetin, luteolin, and kaempferol), steroids, terpenoids, glycosides, sesquiterpenoids, essential oils, polysaccharides, and a mixture of fatty acids [13]. Syringin, berberine, and rumphioside-I were the alkaloids studied, and in silico analyses showed that they significantly inhibited insulin receptor substrate-1 (IRS-1) and IRS-2 receptors by the antagonistic ligand. When docked against the human androgen receptor 1E3G, BER and PAL performed better than other compounds. Isoquinoline alkaloids (BER and PAL) may have functions against a variety of disorders; for example, virtual screening of an alkaloid from the stems of TC against PCOS led to a scientific evaluation [14]. Furthermore, BER effectively restored serum hormone levels and reduced insulin resistance. Apoptosis and morphological damage to the ovaries were both repaired by BER therapy. BER’s effects on granulosa cell proliferation and death were mitigated by blocking the PI3K/AKT pathway [15]. There is hope that these medications will have promising outcomes in PCOS after they have undergone comprehensive preclinical and clinical testing [16].

The Food Safety and Standards Authority of India (FSSAI) considered the root and stem of *Tinospora cardifolia* as a nutritional supplement in the form of powder, decoction, sattva (satva), and extract in a dose varying from 0.5 to 10 g [17]. The primary purpose of conducting this study was to develop extracts, satva, and oil of TC as recommended by FSSAI and examine their effects on DHEA-induced PCOS-related abnormalities such as insulin resistance and sensitivity, glucose tolerance, menstrual disturbance, lipid profile, and microscopic and morphological changes in the ovaries in a mice model, along with elucidating the mechanism of action.

## 2. Materials and Methods

### 2.1. Plant Collection and Authentication

The plant specimens were obtained from the herbal garden of the institute and near the campus and authenticated at HNBG University, Srinagar, Uttarakhand, India (ref.: Plant Taxn/356/2822-A).

### 2.2. Reagents and Chemicals

Insulin testing kits were provided by Wuhan Fine Biotech Co., Ltd., Wuhan, China (batch: M0260H030). Cholesterol and triglyceride testing kits were purchased from Transasia Bio-medicals Ltd., Mumbai, India. Progesterone, testosterone, estradiol, LH, and FSH levels were estimated using ELISA kits purchased from Abbott Laboratories, Abbott Park, IL, USA.

### 2.3. Instruments

The following instruments were used to conduct this research: hemoglucometer (Dr. Morepen Glucose Monitor-Gluco One BG03, Gurugram, India); rotary microtome; Kwality fluorescent microscope with camera, Chandigarh, India; biochemical analyzer for lipid profiles; microplate reader for ELISA tests (Micro lab, New Delhi, India).

### 2.4. Preparation of Hydroalcoholic Extract

TC hydroalcoholic (HA) extract was prepared from stems collected and sterilized. The stems were trimmed into smaller bits and air-dried in the shade and then powdered in a steel blender. For seven days, with occasional shaking and warming, 1 kg of powdered TC stems was immersed in a hydroalcoholic solution (70:30) in a 5 L flat-bottomed flask and cold-extracted. After seven days, the mixture was filtered through a Buchner funnel to create a clear filtrate. Then, the filtrate was evaporated using a water bath and rotary evaporator at 60–70 °C. After evaporation, the hydroalcoholic extract was completely dry and ready for use [18].

### 2.5. Formulation of Guduchi Satva

The fresh TC stems used to make guduchi satva (water extract by following FSSAI/Ayurvedic-approved method of preparation) were washed well with clean water, then chopped into pieces 1 to 2 inches in length, crushed into a coarse, slimy mass, and soaked in water for an entire day. It was carefully macerated and filtered through four layers of muslin the next day. After allowing the filtrate to settle for as long as five hours, the supernatant liquid was skillfully drained, and the starchy sediment was scraped into a tray. A white crystalline powder was made by lyophilizing the starchy material at −55 °C; this was then placed in an airtight container until pharmacological evaluation could be undertaken [19].

### 2.6. Isolation of Essential Oil

The leaves of TC were harvested and then washed with running water to obtain essential oil. The essential oil was obtained by subjecting 100 g of newly harvested TC leaves to a Clevenger apparatus for hydrodistillation for a period of four hours. The hydrodistillation process resulted in an average yield of oil, which was reported [20].

### 2.7. Animal Handling and Groups

Swiss albino female mice that were 6 weeks old with a body weight of 30–40 g were purchased from NIB, Noida–India. After a week’s time for acclimatization, the animals were used for experimentation. All 54 female mice were separated into 9 groups of 6 mice each and treated every day for 20 days. DHEA of 6 mg/100 g/day BW diluted in 0.1 mL sesame oil was given subcutaneously. The acute toxicity study was carried out as per the OECD guidelines [21] and the recently published study [22]. The oral maximum dose of 2000 mg/kg did not cause any toxicity signs. Therefore, 10% of the dose was considered optimal, i.e., 200 mg/kg/day, and its double, 400 mg/kg/day, was considered as the maximum dose and used for this study. Group I: Normal group was administered 0.1 mL sesame oil and 100 μL 0.5% methylcellulose s.c.; Group II: Disease control (DHEA + 100 μL 0.5% methylcellulose); Group III: Positive control (250 mg/kg/day metformin mixed in 0.5% methylcellulose oral + DHEA); Group IV: TC-satva-treated group, 200 mg/kg/day oral + DHEA; Group V: TC-satva-treated group, 400 mg/kg/day oral + DHEA; Group VI: TC-oil-treated group, 200 mg/kg/day oral + DHEA; Group VII: TC-oil-treated group, 400 mg/kg/day oral + DHEA; Group VIII: TC-HA-extract-treated group, 200 mg/kg/day oral + DHEA; Group IX: TC-HA-extract-treated group, 400 mg/kg/day oral + DHEA.

### 2.8. Establishment of PCOS Model

Oral administration of TC preparations (satva, oil, and HA extract) began 2 h before the first DHEA dose and continued for 20 days to assess the preventative benefits of TC extracts in PCOS. The animals were weighed on days 1 and 21. According to a group of researchers [7], blood glucose levels were checked on days 1 and 21. On day 20 of the study, an Oral Glucose Tolerance Test (OGTT) was carried out. Measurements of insulin, testosterone, progesterone, estradiol, cholesterol, LH, FSH, and triglycerides were taken from the animals’ blood at the conclusion of the study. The ovaries were removed for histological examination.

### 2.9. Estrous Cycle Monitoring and Vaginal Smear Test

To measure the variations in the estrous cycle of the test mice, vaginal lining smears were collected. There are four stages to the estrous cycle: estrus, diestrus, metestrus, and proestrus. After flushing the mice’s vaginas with 0.2 to 0.3 mL of normal saline using a micropipette, we aspirated vaginal fluid to analyze the estrous cycle. Droplets of this cell solution were placed on slides under coverslips and observed under a microscope with 10× and 50× lenses to identify methylene blue-stained cells. The presence of cornified epithelial cells indicates the estrous phase; the presence of mucus and leukocytes confirms the diestrus stage; the presence of epithelial cells, leukocytes, and cornified cells with nuclei indicates the metestrus phase; and epithelial cells with nuclei with few leukocytes indicate the proestrus stage [23].

### 2.10. Change in Body Weight

Regular changes in the body weight of female mice were monitored on day 1, day 14, and day 21 [24].

### 2.11. Measurement of Fasting Blood Glucose (FBG) and Oral Glucose Tolerance Test (OGTT)

On day 20, for the OGTT measurement, the mice were given glucose orally at a rate of 2 g/kg of body weight after they had been deprived of food for a period of six hours. A hemoglucometer (Dr. Morepen Glucose Monitor-Gluco One BG03, Gurugram, India) was used to measure the amount of glucose in the blood at 60 and 120 min. The fasting blood glucose was measured on days 1 and 21 [25].

### 2.12. Blood Collection, Collection of Plasma, and Detection of Biochemical Indexes

On day 21 of the study, retro-orbital and cardiac blood samples were collected and well preserved in BD Vacutainers with potassium EDTA to avoid clotting until use. Hemolysis, clotting, or clot-forming samples were discarded. Following the collection of blood on the 21st day, serum samples were taken following the centrifugation of blood at 3000 rpm and 4 °C for 10 min. These samples were then stored in a deep refrigerator at a temperature of −80 °C [26]. We assessed the levels of cholesterol and triglycerides by using a biochemical analyzer, in addition to fasting insulin (FINS) levels, followed by the calculation of HOMA-IR, HOMA-Beta, and QUICKI. Serum estradiol, progesterone, and testosterone levels were also estimated according to the manufacturer’s instructions using a microplate reader. There were limited serum samples available for LH and FSH estimation. Therefore, the serum samples of two animals, 150 µL each, were pooled into one, and LH and FSH were estimated using the ELISA test kit [24].

### 2.13. Measurement of Weight, Size, and Morphological Changes in the Ovaries

After blood collection, animals were euthanized with an overdose of ketamine and their ovarian weight, size, and morphology were assessed [27].

### 2.14. Histological Examination

After 21 days, the mice had their ovaries removed and preserved in 10% formalin. The previously reported method was followed for the histological examination. In brief, blocks were serially sectioned at 5 µm thickness using a microtome after undergoing xylene deparaffinization, rehydration, hematoxylin and eosin staining, dehydration, clearing, and mounting on DPX (dibutylphthalate polystyrene xylene) beneath glass coverslips. After that, we observed the slides under a microscope [26].

### 2.15. Severity of PCOS

Based on previous literature [28] and our own understanding, we propose the following method of calculating the severity score of PCOS in mice and categorize it as nil, mild, moderate, severe, and very severe.

$$PCOS\;Severity\;Score=\left(Presence\;or\;Absence\;of\;Oocyte+Number\;of\;Cystic\;follicles\right)\times \;\frac{Ovary\;diameter\;\left(\mathrm{mm}\right)+Ovarian\;weight\;\left(\mathrm{mg}\right)/10}{10\times (Insulin\;\left(\frac{\mathrm{IU}}{\mathrm{mL}}\right)+Testosterone\;\left(\frac{\mathrm{nmol}}{L}\right))}$$

where the presence of oocytes = 0 and the absence of oocytes = 1.

Then, we categorize the severity of PCOS based on the numerical score:

Severity score: ≥10 (very severe), 7–10 (severe), 4–7 (moderate), 1–4 (mild), <1 (nil).

### 2.16. Statistical Analysis

Statistical analysis was performed, and the data are presented as mean ± SEM. The unpaired T test was used to analyze the categorical data, while the paired T test, one-way analysis of variance (ANOVA), and Dennett’s multiple comparisons test were used to analyze the continuous data. Statistical significance was assumed when the *p*-value was less than 0.05. The results are indicated in figures as * *p* < 0.05, ** *p* < 0.01, and *** *p* < 0.001 compared to disease control mice.

## 3. Results

### 3.1. Estrous Cycle Determination

**Figure 1:**
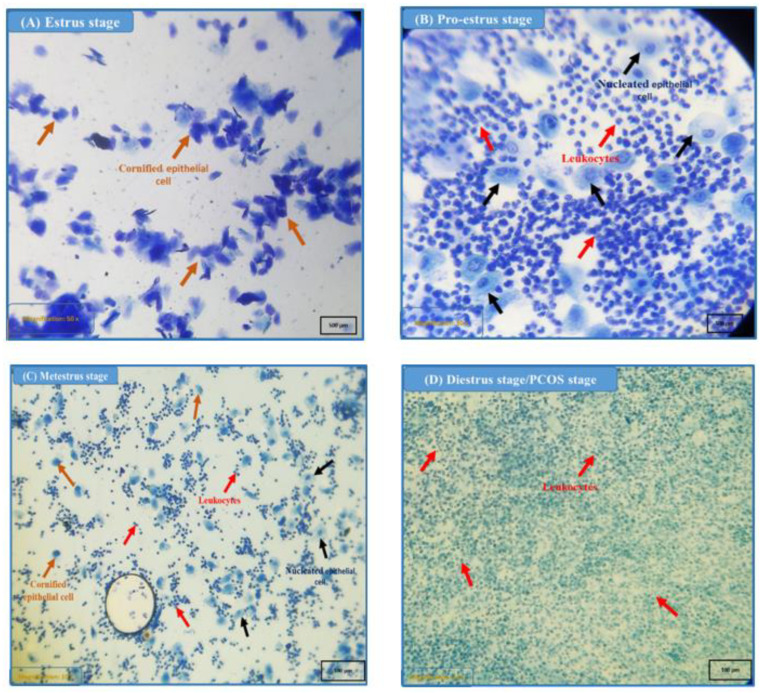
The different stages of the estrous cycle were estimated in normal healthy mice. (**A**) In the estrus stage, predominantly cornified epithelial cells (brown arrow) are present. (**B**) The pro-estrus stage is characterized by nucleated cornified epithelial cells (black arrow) with leukocytes (red arrow). (**C**) The metestrus stage contains cornified epithelial cells (brown arrow), leukocytes (red arrow), and nucleated epithelial cells (black arrow). (**D**) In the diestrus stage or PCOS stage, predominantly leukocytes (red arrow) are present.

In Figure 1, it is observed that Group I had all four phases of a normal estrous cycle throughout the study, showing a healthy mouse’s estrous cycle. Group II animals with PCOS had delayed estrous cycles that lingered in diestrus/PCOS. As most vaginal smear cells were leukocytes, Group III (Metformin) remained in diestrus. Groups IV, V, VI, VII, VIII, and IX exhibited the presence of cornified epithelial cells.

### 3.2. Body Weight

**Figure 2:**
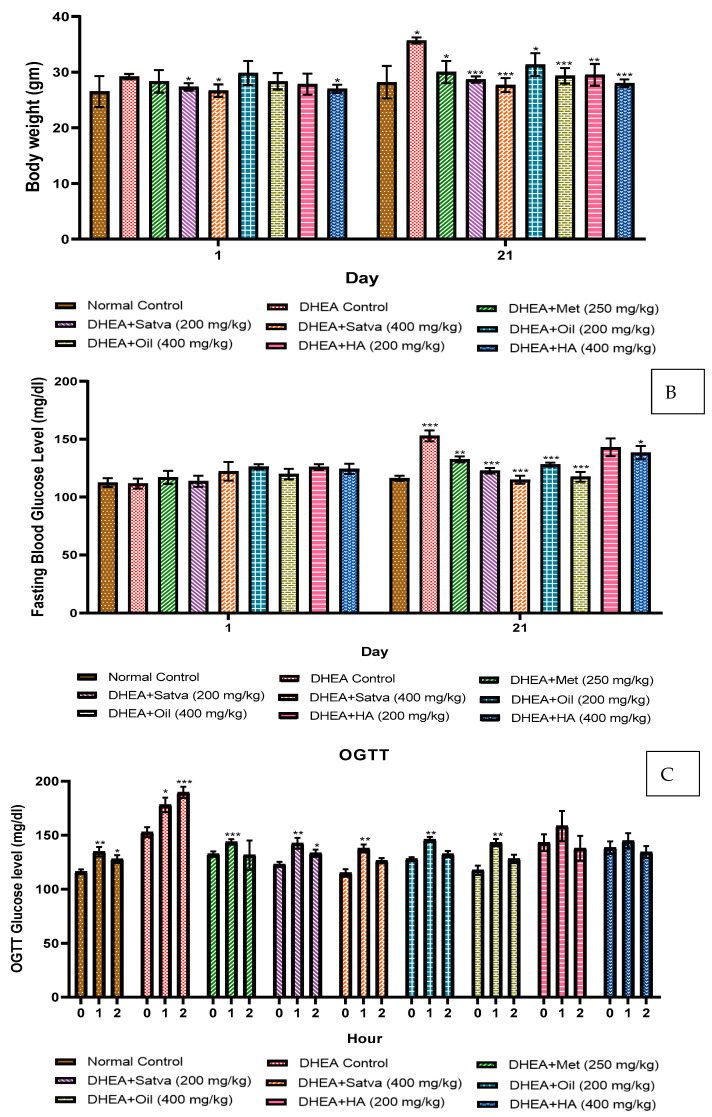
Effects of Tinospora cordifolia extract treatment in DHEA-induced PCOS mice on body weight (**A**), fasting blood glucose (**B**), and OGTT (**C**) measurement. Every value is presented as a mean along with standard error. Data were analyzed by ANOVA followed by DMCT. In all groups, 0 h of OGTT was compared with 1 and 2 h of OGTT. * *p* < 0.05, ** *p* < 0.01, and *** *p* < 0.001.

Figure 2A shows that DHEA-treated mice gained considerably more weight by day 21 than their normal control counterparts did (*p* < 0.05). Mice that were given TC satva had a significant (*p* < 0.001) decrease in BW compared to those given a placebo. The treatment group receiving 400 mg/kg of TC oil and HA extract also lost significantly more weight than the DHEA control group (*p* < 0.01).

### 3.3. Fasting Blood Glucose Level

As presented in Figure 2B, on day 1, the blood glucose level was normal in all groups. On day 21, the DHEA group had highly significantly (*p* < 0.001) increased BG levels when compared to normal control mice. Highly significant (*p* < 0.001) reductions in BG were observed in groups of mice treated with TC satva and oil on day 21 compared to the negative control group. Treatment with metformin significantly (*p* < 0.05) reduced the DHEA-induced increased blood glucose level but not at the level of TC treatment groups.

### 3.4. Oral Glucose Tolerance Test

As shown in Figure 2C, on day 20, 1 h OGTT of TC satva and oil treatment significantly (*p* < 0.01) raised glucose levels compared to 0 h OGTT of TC satva and oil, but it was normal after 2 h OGTT of TC satva and oil. When compared to the 0 h OGTT of the DHEA group, the 1 h OGTT and 2 h OGTT of Group II demonstrated a significant rise in BG levels. There was no alteration in OGTT for the normal control group. The 2-h OGTT results indicate that TC treatment for 20 days significantly improves glucose tolerance in mice.

### 3.5. Hormonal Profile

**Figure 3:**
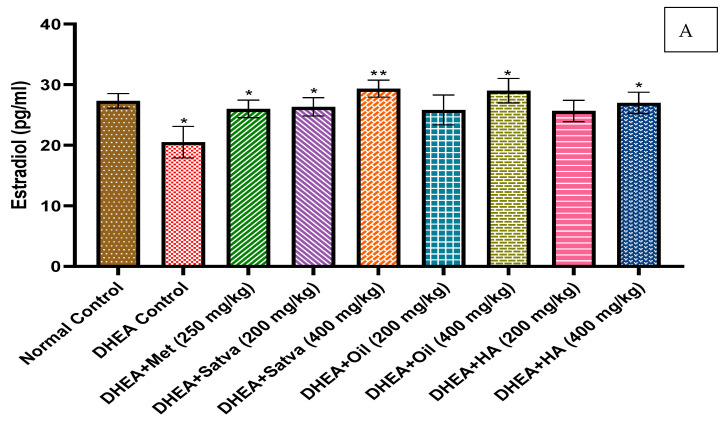

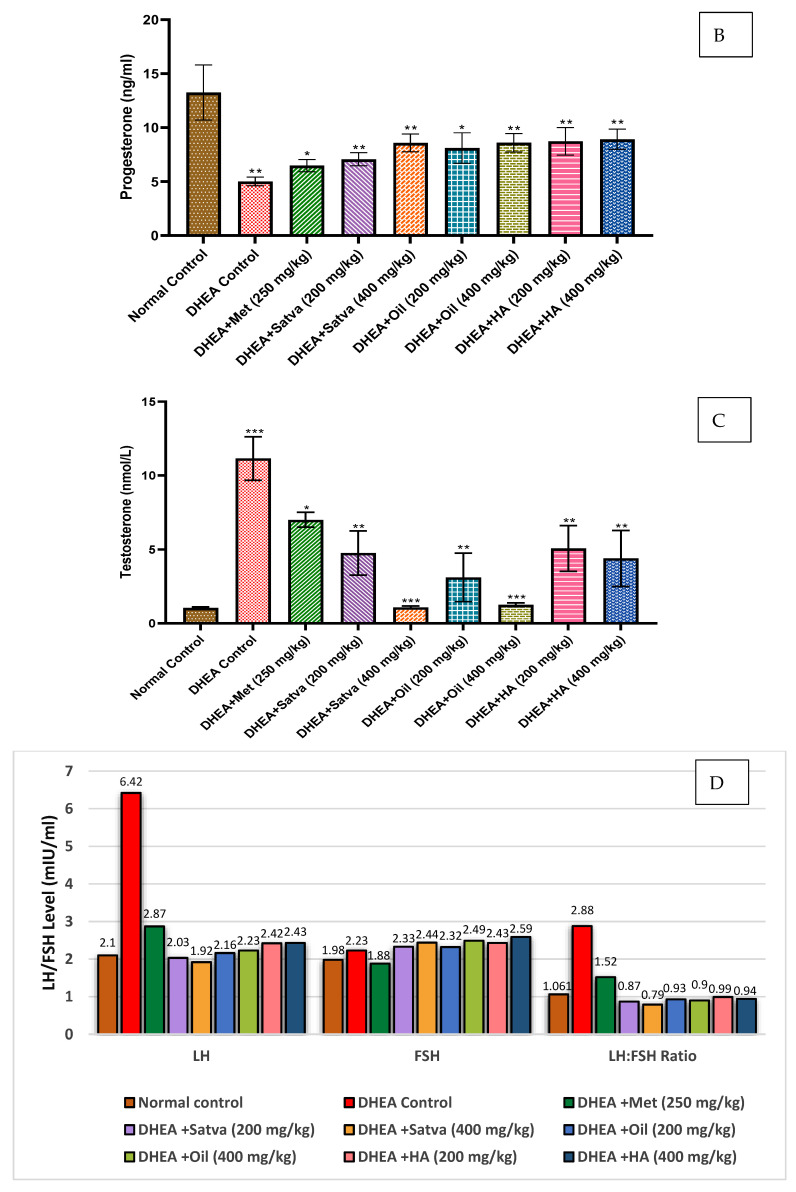
Treatment with *Tinospora cordifolia* extracts was given to mice that had DHEA-induced PCOS, and the levels of estradiol (**A**), progesterone (**B**), testosterone (**C**), and LH/FSH (**D**) were measured. Every value is presented as a mean along with standard error. Data were analyzed by ANOVA followed by DMCT. * *p* < 0.05, ** *p* < 0.01, and *** *p* < 0.001.

As presented in Figure 3A,B, the estradiol and progesterone levels were observed to be considerably (*p* < 0.05 and *p* < 0.01) lower in DHEA-treated mice compared to Group I. However, testosterone levels (Figure 3C) were found to be statistically (*p* < 0.001) greater. In all TC treatment groups, there was a decrease in serum testosterone, but the highest doses of satva and oil brought it down to nearly normal levels. Metformin counteracted the effect that DHEA had on estradiol (*p* < 0.05). When compared to Group II (20.5 ± 2.59), the maximum dose (400 mg/kg) of TC satva, oil, and HA extracts significantly increased estradiol levels (TC satva (29.33 ± 1.406), oil (29 ± 2.017), and HA extracts (27 ± 1.75)). Treatment with TC extract prominently raised (*p* < 0.01) serum progesterone levels when compared to the placebo group.

### 3.6. LH and FSH Level

As shown in Figure 3D, DHEA treatment resulted in greater increases in levels of FSH (2.23 mIU/mL) and LH (6.42 mIU/mL) in mice than in the controls (FSH (1.98 mIU/mL) and LH (2.1 mIU/mL)). LH and FSH levels were found to be lower in mice that were given TC extracts compared to those given a placebo for their condition. The LH/FSH ratio of DHEA groups was increased (2.87) as compared to normal mice (1.06). In all TC extract treatment groups, the FSH was higher than LH. However, metformin treatment was slightly significant in reversing the effects of DHEA.

### 3.7. Lipid Profiles

**Figure 4:**
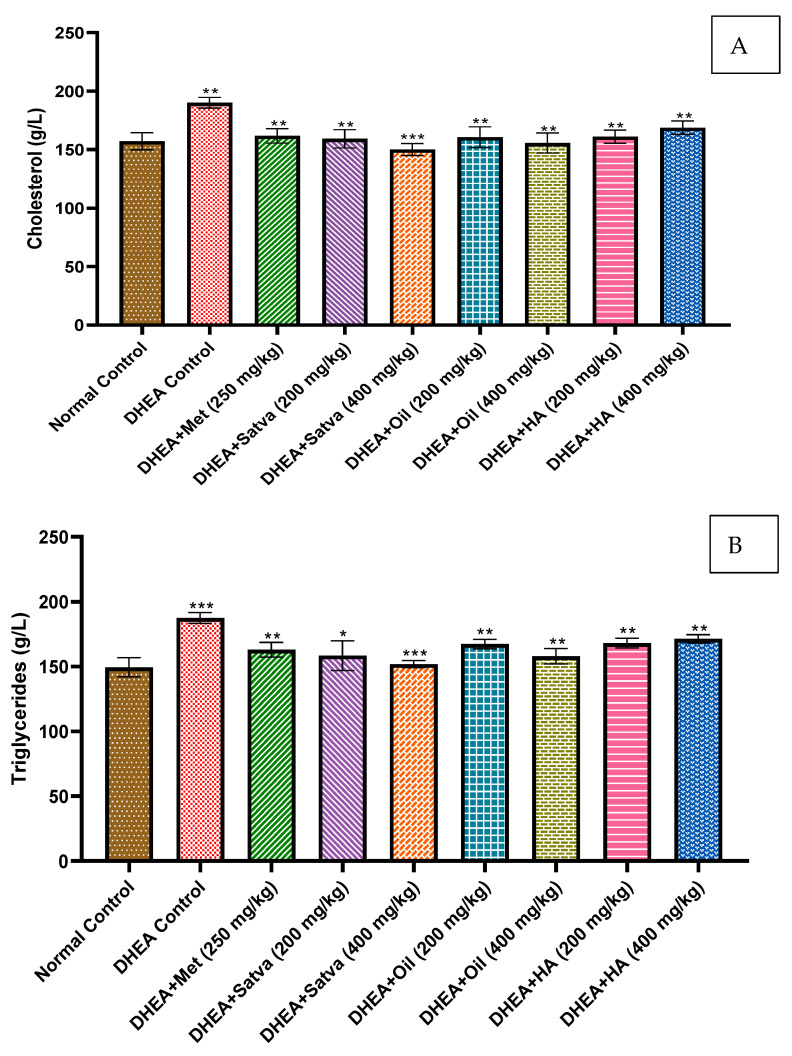
Effects of *Tinospora cordifolia* extract treatment in DHEA-induced PCOS mice on cholesterol (**A**) and triglyceride (**B**) levels. Every value is presented as a mean along with standard error. Data were analyzed by ANOVA followed by DMCT. * *p* < 0.05, ** *p* < 0.01, and *** *p* < 0.001.

Figure 4A,B shows that the DHEA group had significantly higher levels of cholesterol and triglycerides than the control group (*p* < 0.01). Metformin reduced blood cholesterol levels significantly (*p* < 0.01) in mice when administered at a dose of 250 mg/kg. All groups treated with TC extracts had significantly decreased serum cholesterol and triglyceride levels compared to the DHEA group (*p* < 0.01 to *p* < 0.001).

### 3.8. Insulin Profile

**Figure 5:**
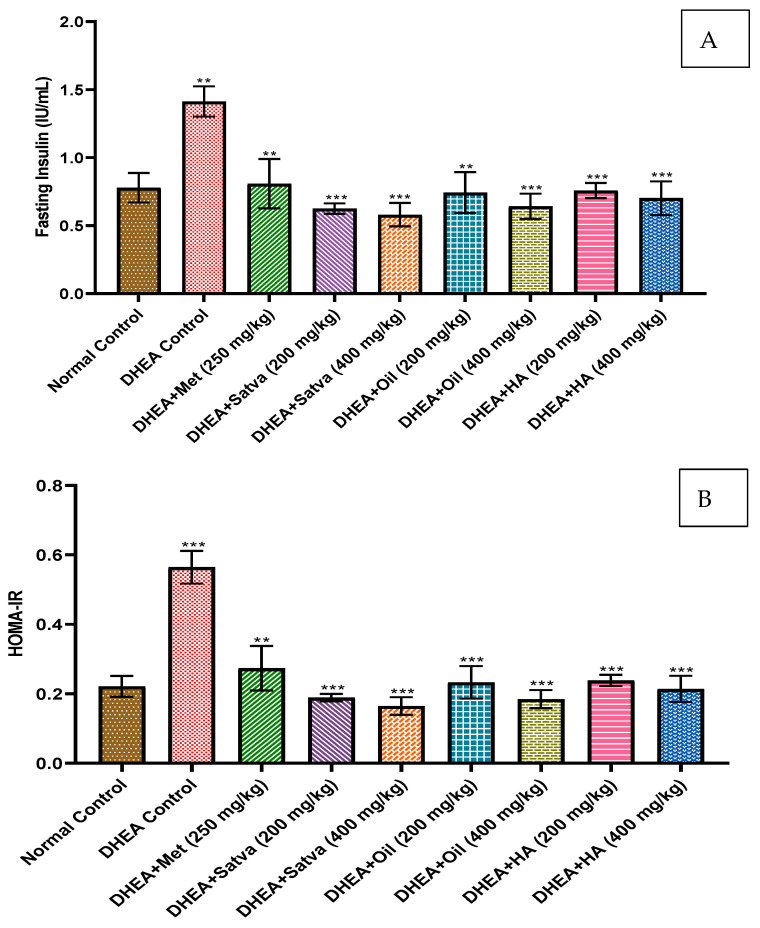

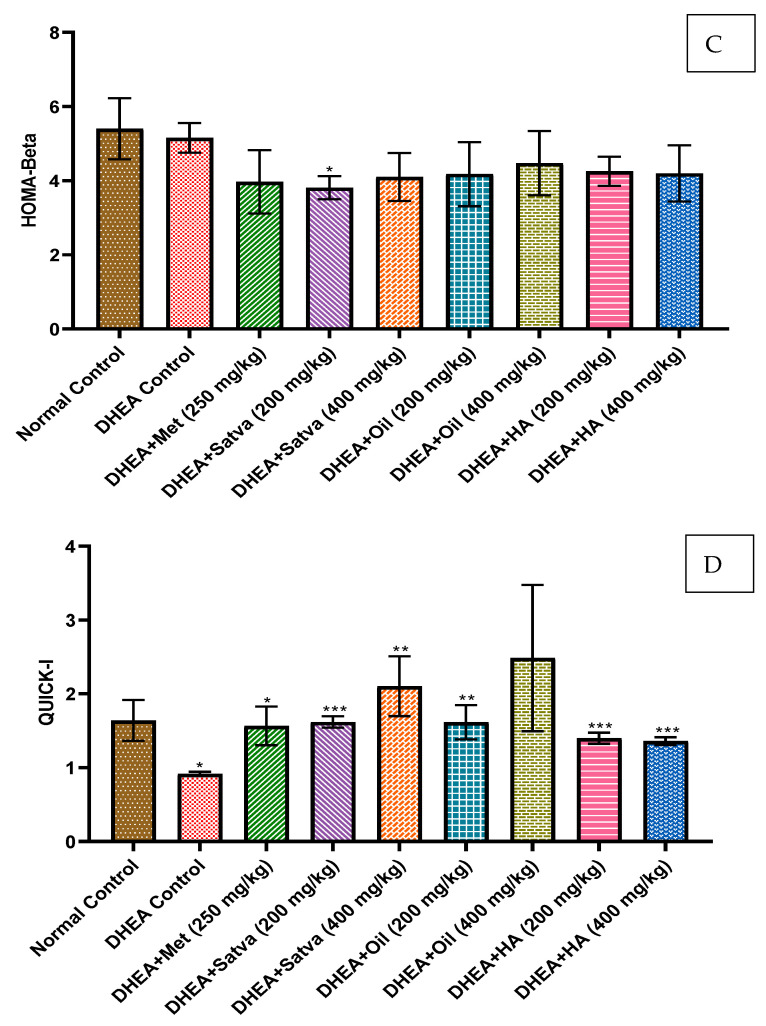
Effects of *Tinospora cordifolia* extract treatment in DHEA-induced PCOS mice on fasting insulin levels (**A**), HOMA-IR (**B**), HOMA-Beta (**C**), and QUICKI levels (**D**). Every value is presented as a mean along with standard error. Data were analyzed by ANOVA followed by DMCT. * *p* < 0.05, ** *p* < 0.01, and *** *p* < 0.001.

DHEA-treated mice had significantly increased insulin levels (Figure 5A) compared to controls (*p* < 0.01). Serum insulin was significantly decreased (*p* < 0.001) in the TC-treated group of DHEA-pretreated animals. Similarly, the HOMA-IR index (Figure 5B) was highly significantly (*p* < 0.001) reduced by TC treatment in mice. Treatment with TC oil and HA extract failed to stimulate beta cells in DHEA-treated mice as indicated in the HOMA-Beta index (Figure 5C). A dose-independent effect of TC on DHEA-induced QUICKI (Figure 5D) was observed and was highly significant (*p* < 0.01 and *p* < 0.001) with HA extract, oil, and satva compared to the negative control. However, the lower doses of TC satva, oil, and HA extracts significantly improved QUICKI.

### 3.9. Ovarian Size and Weight

**Figure 6:**
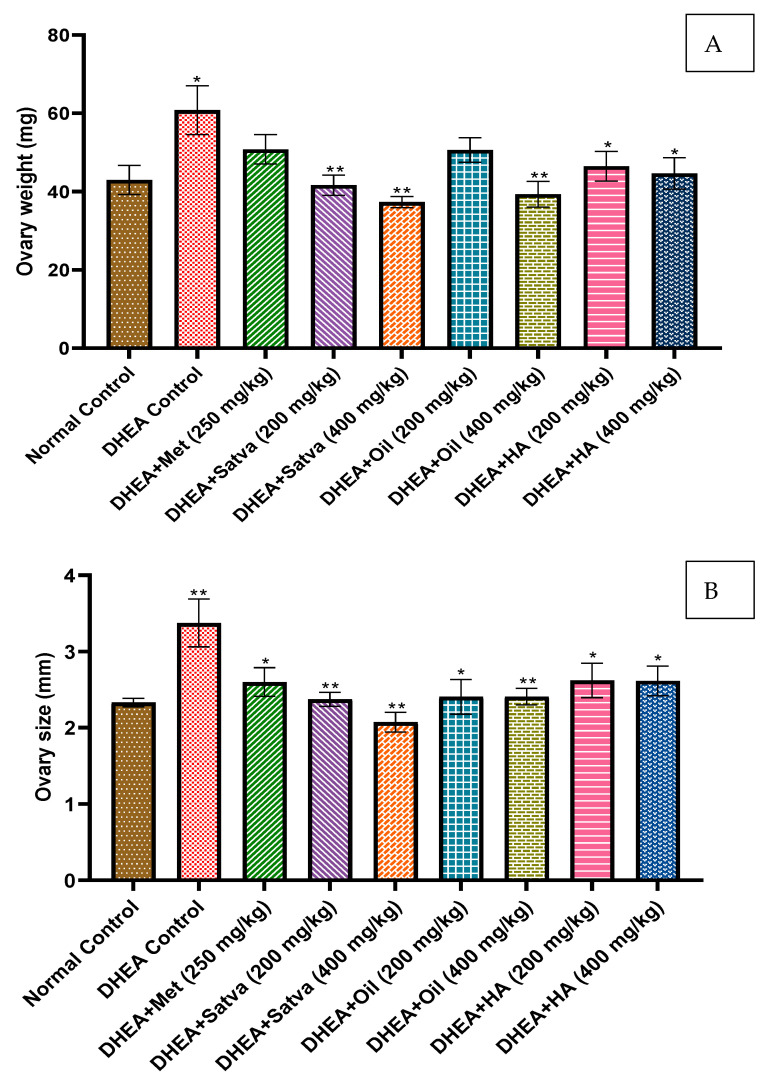
The effect that treatment with Tinospora cordifolia extracts had on ovary weight (**A**) and size (**B**). Every value is presented as a mean along with standard error. Data were analyzed by ANOVA followed by DMCT. * *p* < 0.05, ** *p* < 0.01.

In this study, we used the right ovary of each mouse for morphological examination and the left ovary for histopathological study. The ovary diameter (Figure 6B) of DHEA-treated mice was substantially (*p* < 0.01) larger than that of the control group. This demonstrates that ovarian hypertrophy is brought about by DHEA therapy. The ovarian diameter was found to be significantly (*p* < 0.01) reduced after TC preparation therapy. Similar results were seen with metformin treatment. The weight of the ovaries, as shown in Figure 6A, was also computed in this study. DHEA treatment significantly (*p* < 0.05) increased the weight of the ovaries compared to control mice. Treatment of TC for 21 days did not allow the weight of the ovaries to increase, and they maintained almost a normal weight. TC treatment reduced the DHEA-induced ovarian hypertrophy and ovarian weight very significantly (*p* < 0.01).

### 3.10. Morphological Observation of Ovaries

**Figure 7:**
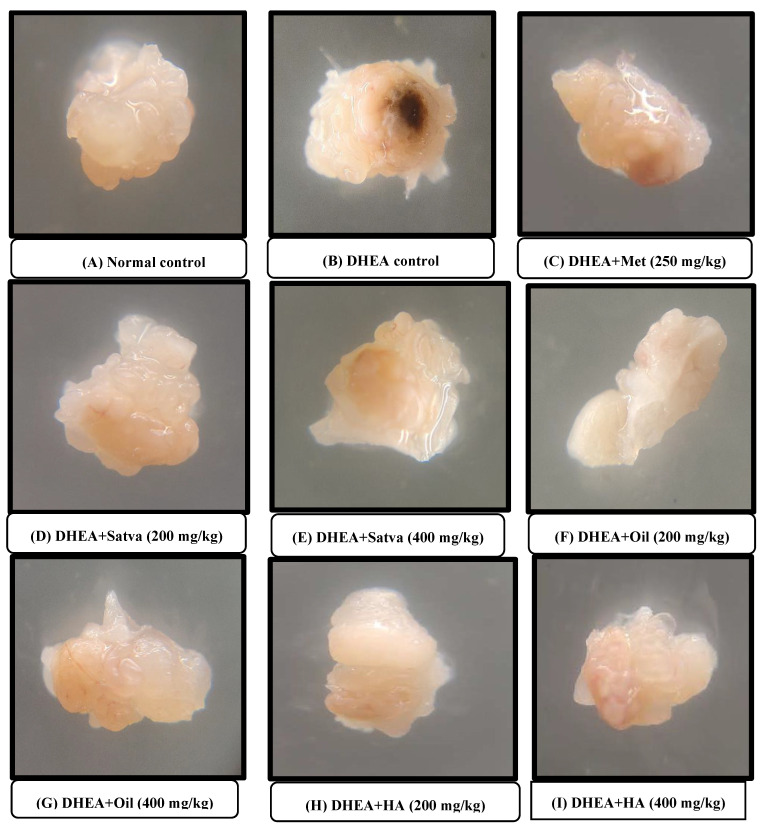
Effects of *Tinospora cordifolia* extract treatment in DHEA-induced PCOS mice on morphological examination of the ovaries. Histological analysis of normal ovaries compared with PCOS and ovaries treated with TC preparations. (**A**) Normal control, (**B**) DHEA treated, (**C**) positive control treated by metformin, (**D**) TC satva 200 mg/kg treated, (**E**) TC satva 400 mg/kg treated, (**F**) TC oil 200 mg/kg treated, (**G**) TC oil 400 mg/kg treated, (**H**) TC HA 200 mg/kg treated, (**I**) TC HA 400 mg/kg treated.

Observation of the ovaries with the naked eye showed that the DHEA treatment increased the ovary volume and weight and led to the appearance of black spots and granular changes, as demonstrated in Figure 7. Treatment with metformin reduced the black spots and granulation, but not as good as in the TC-treated groups. Treatment of mice with TC satva and oil in DHEA-induced PCOS resulted in normal ovaries. Treatment with TC HA extract also significantly reduced the alterations in the appearance of ovaries.

### 3.11. Histopathological Examination of the Ovaries

**Figure 8:**
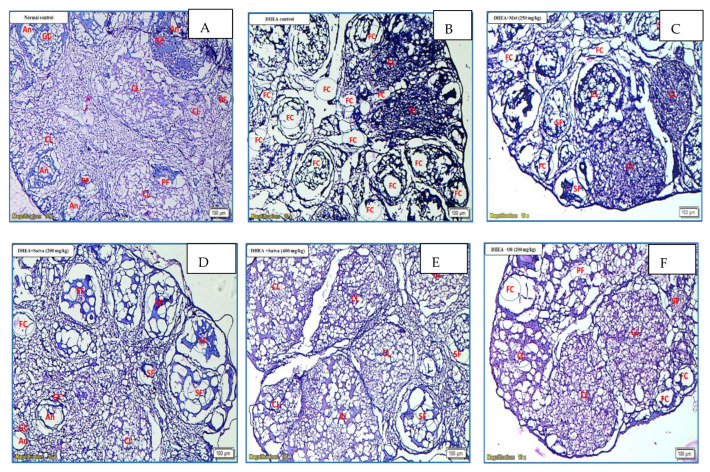

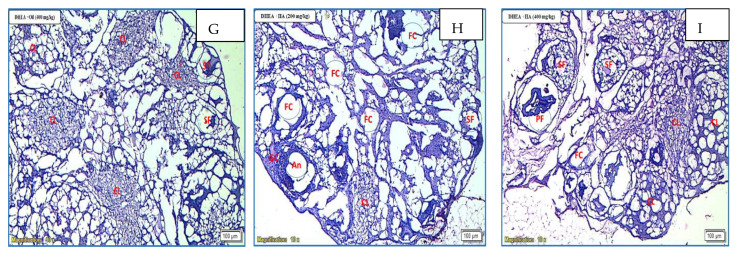
Effects of TC extracts on histopathology of DHEA-induced PCOS ovaries in mice. Comparative histological examination of normal and PCOS ovarian tissue, as well as ovarian tissue that was treated with TC formulations. Hematoxylin and eosin (H&E) staining, 10× magnification, 100 µM scale, as detailed in Section 2. (**A**) The untreated normal control, (**B**) the treated positive control with DHEA, (**C**) the treated positive control with metformin, and (**D**–**I**) mice treated with TC satva 200, TC satva 400, TC oil 200, TC oil 400, TC HA 200, and TC HA 400 mg/kg, respectively. AF is short for antral follicle, FC stands for cystic follicle, CL stands for corpus luteum, GC refers to granulosa cell, PF stands for primary follicle, and SF stands for secondary follicle.

As presented in Figure 8, oocytes were found in the normal control group’s ovary follicles. These oocytes were surrounded by theca cells and granulosa cells. Theca cells are polarized mesenchymal cells having a spindle-like form and typically have two to three layers of thickness. These cells were present outside of the follicle-associated basal lamina. An immature interstitial compartment was found outside of the theca layer. The cells that made up this compartment were formed from steroidogenic theca cells that had been left over from atretic follicles. After TC therapy, PCOS-induced mice ovaries showed a larger number of cystic follicles and fewer preantral follicles, corpus luteum, stomal nodules, stromal hyperplasia, and antral follicles compared to controls. In addition, during the ovarian histopathology study after TC treatment, there were no corpora lutea present, which is proof that ovulation had not taken place. Ovary sections from a TC-satva-treated mouse demonstrated the presence of a medulla, a follicular cyst, and a Graafian follicle. Medulla, cortex, and follicular cysts were seen in the metformin-treated group. When the organ was cut transversely, the components of the normal control group’s ovary could be seen, each with a clearly defined structure. The cortex of the ovary revealed different stages of follicle development. There were unruptured primordial follicles, as well as primary follicles, secondary follicles, and Graafian follicles. The blood arteries were normal, and the surface epithelium was bordered with low cuboidal to flattened cells. The stroma is made up of theca cells that range in shape from round to spindle. The mice that were given DHEA showed stromal nodules and follicular cysts coupled with infrequent Graafian follicles. Additionally, these mice had multiple follicular cysts linked with hyperplasia of theca cells. The follicular cysts, medulla, and Graafian follicles were all visible in the ovaries of mice that had been treated with TC oil. The ovary of a mouse that had been treated with TC hydroalcoholic extract exhibited a medulla, cortex, and follicular cyst. Because of this, the findings suggest that there was an improvement in histopathological symptoms of the mice that were given TC satva and oil as a treatment and that these mice ovulated.

### 3.12. Severity Score Measurement

**Figure 9:**
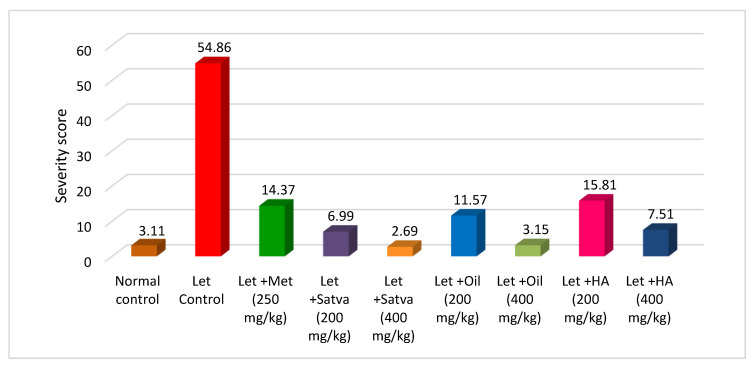
The effect that treatment with Tinospora cordifolia extracts had on severity score for PCOS.

In this 21-day study, we estimated the severity score for PCOS (Figure 9). The severity in DHEA-treated mice was higher (54.86) than in the control. A highly significant decrease in severity was observed with TC preparation treatment. The results with metformin treatment were not more significant. Treatment of mice with TC satva and oil in DHEA-induced PCOS resulted in highly reduced severity. Treatment with 400 mg/kg TC HA extract also significantly reduced the severity score.

## 4. Discussion

Metabolic instability is commonly connected with polycystic ovarian syndrome, a common reproductive condition. Insulin resistance, diabetes mellitus, and dyslipidemia are all conditions that are more likely to occur in women with PCOS. Obesity, insulin resistance, genetic predisposition, inflammation, and oxidative stress are just some of the theories proposed to explain PCOS [29]. PCOS is a complicated illness, and women with it often experience ovarian and neuroendocrine dysfunction [30,31]. Insulin has been shown to increase androgen release from ovarian stromal and thecal cells in in vitro studies [24]. Hyperandrogenism is caused by an increase in ovarian androgen production due to insulin resistance and hyperinsulinemia in women with polycystic ovary syndrome. Although the exact cause of PCOS in women is unknown, its primary characteristics can be replicated in a mouse model using DHEA [32,33,34].

Being a potent anti-inflammatory herbal drug, it was anticipated that TC extracts would help in improving insulin balance and decrease the risk of developing ovarian cysts. As an agent for rejuvenation, it may stimulate all the body tissues and improve metabolism naturally [12]. We examined the effects of TC extracts on body weight, ovarian weight, insulin sensitivity, and ovarian shape. In this study, DHEA was given at a dose of 6 mg/100 g/day for 21 days to mimic the reproductive characteristics matching the PCOS condition. Increases in body weight, ovarian weight, glucose, LH, testosterone, and insulin resistance were all indicators of compromised reproductive health in DHEA-treated groups compared to controls. It was found that the extracts restored normal body weight, reduced PCOS-associated dyslipidemia, and reduced insulin resistance. It also regulated the level of hormones such as LH, FSH, estradiol, progesterone, and testosterone. The morphological and histopathological studies proved that the extracts inhibit changes in the cellular, tissue, and organ systems of the body.

In this study, the PCOS group animals’ estrous cycles (consisting of estrous, proestrus, metestrus, and diestrus phases) were significantly arrested and delayed, with the diestrus phase lasting significantly longer than the other three [35], similar to our study results where most vaginal smear cells of negative control groups had leukocytes. The normal control group (Group I) showed all phases of a normal estrous cycle, i.e., estrous, proestrus, metestrus, and diestrus. Group III (Metformin) remained in the diestrus phase for a long period. Groups IV, V, VI, VII, VIII, and IX exhibited the estrous phase of the cycle having cornified epithelial cells. Restoring normal circulation concentrations of testosterone, estrogen, progesterone, and gonadotrophins may be related to TC’s restorative impact on the mouse estrous cycle, as presented in this study. Ovarian functions, such as follicular maturation and hormonal imbalance, are believed to be normalized by a regular estrous cycle, which is regulated by these hormones [36].

On day 21, DHEA-treated mice had increased body weight compared to the normal control group. PCOS and weight gain were associated with DHEA injections. There is a lot of evidence linking PCOS and being overweight [27]. In this study, the BW of mice treated with DHEA was significantly increased due to abdominal fat compared to the normal mice [37]. An increase in growth hormone production from the anterior pituitary gland, which in turn stimulates the release of insulin-like growth factors (IGFs) I and II, may have occurred due to the treatment’s effect on the metabolic pathway [38]. This hormone is essential for promoting the cellular absorption of amino acids, regulating lipolysis, and promoting skeletal muscle development. This confirms what previous studies found, which was a considerable rise in female mice’s body weight through increased protein synthesis and slowed protein breakdown with the help of IGF I and II. In contrast, satva significantly lowered the BW of mice by the process of restoration when compared to the control group of animals. The treatment group receiving 400 mg/kg of TC oil and HA extract also lost significant extra weight that was gained due to DHEA (*p* < 0.01). After 21 days of therapy, the mice’s body weight had approximately reached that of the normal mice. This result agrees with what has been seen before because TC treatment controls cellular homeostasis, energy metabolism, loss of lipids, and hypoglycemia [13,37].

On day 1, blood glucose levels were normal in all groups. On day 21, the experimental animals in the DHEA-only-treated group had raised blood glucose levels compared to the normal control group. This study’s findings that DHEA-induced PCOS mice exhibit hyperglycemia are in line with those of earlier research [39]. Compared to a DHEA control group, mice given TC extracts had significantly reduced blood glucose levels. Treatment with TC satva and oil resulting in hypoglycemia may be due to the activation of glucose 6-phosphatase and normalizing levels of liver glycogen, suppression of oxidative stress, inhibition of gluconeogenesis, and inhibition of glycogenolysis [40]. Reported insulin-mimicking and insulin-releasing actions, insulin secretion, glucose uptake, and suppression of peripheral glucose release in hyperglycemia have been found in TC’s isoquinoline alkaloids, including palmatine, jatrorrhizine, and magnoflorine [41,42].

On day 20, 1 h OGTT of TC satva and oil treatment raised glucose levels compared to 0 h OGTT of TC satva and oil, but it was normal after 2 h OGTT of TC satva and oil. Moreover, 1 h OGTT of the DHEA-treated group was increased as compared to 0 h OGTT of the DHEA group, but after 2 h, there was a highly significant rise in blood glucose levels. This shows that there was an insufficient amount of insulin released upon loading the glucose. To combat diabetes, decreasing hepatic phosphorylase activity and increasing glucose absorption by peripheral tissues and organs, including the liver, may be effective [43], which could be the mechanism of action of TC preparations.

Estradiol and progesterone levels were comparatively lower in the negative control group compared to normal controls. Low progesterone levels cause anovulation. Higher plasma androgen and lower estrogen and progesterone concentrations have been observed in PCOS patients [9]. Hyperandrogenism is caused by the overproduction of ovarian androgens due to insulin resistance with hyperinsulinemia in PCOS females. It is believed that IGF-1 receptors on theca and stroma cells are the mechanism through which insulin regulates ovarian androgen production [44]. The growth hormone IGF-1 promotes granulosa cell aromatase production [45] and works in concert with FSH and LH, regulating the production of aromatase in these cells [24]. In addition, raising androgen levels can increase FSH receptor levels in PCOS patients, leading to a reduction in FSH serum levels via negative feedback. Studies have linked PCOS to insulin resistance due to abnormally high LH or low FSH levels. Both testosterone and LH levels rise when the normal hypothalamic–pituitary–gonadal axis is disrupted, which is what causes the condition [46]. LH decreases progesterone levels and increases androgen levels by stimulating testosterone release from theca cells via the PI3K/Akt pathway. This oversupplies the activity of the 17-a hydroxylase enzyme, catalyzing the conversion of progesterone to androgens [47]. All TC treatment groups saw a decrease in serum testosterone, but the highest doses of satva and oil brought it down to nearly normal levels. Metformin treatment counteracted the estrogenic effects of DHEA. Maximum satva, oil, and HA extracts significantly increased estradiol levels relative to the DHEA-treated group. All treatments with TC extracts resulted in a significant increase in serum progesterone relative to the placebo group. DHEA treatment led to a greater increase in levels of LH and FSH in mice than in the controls. LH and FSH levels were found to be lower in mice given TC extracts compared to those given a placebo for their condition. The LH/FSH ratio of DHEA groups was increased as compared to normal and TC extract treatment groups. In all TC extract treatment groups, the FSH was higher than LH. However, metformin treatment slightly significantly reversed the effect of DHEA. The ratio of LH to FSH dropped significantly after treatment with satva, oil, or hydroalcoholic extracts. The hormone levels of LH and FSH were restored to normal with the use of TC extracts.

Regular treatment of mice with DHEA led to an increase in plasma cholesterol and triglycerides compared to Group I (*p* < 0.01). Biochemical analysis of PCOS patients reveals dyslipidemia, characterized by a lipid profile that is out of whack: low HDL, higher triglycerides (TG), higher total cholesterol, and higher low-density lipoprotein (LDL) [9]. Dyslipidemia, characterized by increased TGs and decreased HDL cholesterol, is commonly observed in PCOS. Dyslipidemia associated with PCOS does not discriminate by weight. The syndrome is associated with a considerable increase in the risk of developing type II DM due to the synergistic effects of obesity and insulin resistance. Patients with PCOS may experience dyslipidemia due to a variety of factors. IR has a significant role by increasing lipolysis and altering the production of hepatic lipase and lipoprotein lipase. There were significant reductions in blood cholesterol and TG concentrations in the TC treatment animals compared to the DHEA control group. The most interesting result was the normalization of TG and cholesterol levels in the animals treated with TC. Previous research has revealed that the lipid-lowering characteristics of TC’s active components, including its alkaloids, glycosides, flavonoids, saponins, and tannins, may be responsible for the regulated BW and lipid profile [13].

DHEA-treated mice exhibited hyperinsulinemia, a marker of insulin resistance, in contrast to the normal control group. Similar to the hyperinsulinemia/hyperandrogenism combination seen in PCOS, insulin resistance can significantly raise levels of circulating proinflammatory cytokines [48]. The symptoms of PCOS, including elevated levels of androgens and irregular or absent menstrual cycles, are likely caused by insulin resistance, which also raises the risk for hyperlipidemia, diabetes mellitus, and cardiovascular disease [49]. Hence, decreasing insulin resistance could be the most important step, and it may also improve reproductive health [24]. In mice treated with DHEA, the administration of TC resulted in a significant decline in blood insulin levels, demonstrating a reversal of insulin resistance. DHEA caused a rise in the HOMA-IR index, which was then reversed by TC therapy. A highly significant reduction in serum insulin levels was observed after TC treatment in the DHEA-pretreated group of mice. Similarly, the HOMA-IR index was reduced by TC treatment in mice. A dose-independent effect of TC on DHEA-induced QUICKI was observed with HA extract, oil, and satva compared to the negative control. However, the lower doses of TC satva, oil, and HA extracts significantly improved QUICKI. The mechanism whereby the active components of TC increase muscle glucose absorption is supported by the Insulin Sensitivity Index QUICKI. Treatment with TC oil and HA extracts demonstrated a dose-dependent improvement in the activity of beta cells, reflected in the HOMA-Beta values [50]. These findings are consistent with earlier findings that TC reduced fructose-induced increases in blood sugar, insulin, and triglycerides. The berberine alkaloid, discovered in the TC plant stem, has been shown to significantly enhance glucose tolerance. Treatment with berberine significantly reduces fasting plasma glucose, improves insulin sensitivity, and decreases LDL-C in type II diabetic rats, demonstrating its potent antihyperglycemic and hypolipidemic effects. The expression of GLUT4 in tissues was significantly increased after being exposed to berberine. Decreased expression of muscle and adipose GLUT4 in diabetic persons is one of the most important reasons for IR, since it is well known that the absorption of glucose into cells depends on the help of membrane GLUT4. In IR animal models, berberine was found to improve insulin sensitivity by activating AMP-activated protein kinase [51].

In silico investigations found that the alkaloids syringin, berberine, and rumphioside-I significantly inhibited IRS1 and IRS2 receptors by acting as antagonistic ligands. Isoquinoline alkaloids (such as berberine and palmatine) play significant roles in warding off a wide range of illnesses. Virtual screening has been used to evaluate the efficacy of an alkaloid extracted from TC stems for the treatment of PCOS [14]. Berberine improved IR and blood hormone levels in PCOS. Berberine therapy also restored ovarian apoptosis and morphological abnormalities. Berberine’s effects on the proliferation of granulosa cells and death were mitigated by blocking the PI3K/AKT pathway [15]. After extensive animal and human studies, it is possible that these drugs may be beneficial in the management of PCOS [16].

In this study, we used the right ovary of each mouse for morphological examination and the left ovary for histopathological study. The diameter of the ovaries of DHEA-treated mice was higher than that of the control group. This shows that DHEA treatment induces hypertrophy of ovaries. Mice given TC oil that had PCOS induced by DHEA appeared to recover to normal health. After treating PCOS mice with TC, follicle numbers were restored and the ovaries were protected from oxidative stress, as was previously described [52]. The weight of the ovary was also computed in this study. DHEA treatment increased the weight of the ovary compared to the normal control group. Consistent with prior observations, a rat model of PCOS induced by DHEA showed that both the animals and the ovaries grew in size [7]. Ovary weight was nearly twice that of the control group after DHEA therapy [36]. Treatment of TC for 21 days did not allow the weight of the ovary to increase and it maintained almost a normal weight. TC treatment reduced the DHEA-induced ovarian hypertrophy and ovarian weight. These are in line with the study in which supplementation with TC extracts has been shown to significantly reduce the risk of morphological abnormalities in the ovaries due to radiation exposure [52]. Hypertrophy, as well as the formation of black patches and granular alterations, were observed in previous research, and these may be seen with the naked eye after DHEA treatment [50]. Treatment with metformin reduced the black spots and granulation but not as well as in TC-treated groups. Treatment of mice with DHEA-induced PCOS using TC satva and oil resulted in normal ovaries. Treatment with TC HA extract also significantly reduced the alteration in the appearance of ovaries. These results are in line with the study in which supplementation with TC extract significantly reduced the risk of morphological abnormalities in the ovaries due to radiation exposure [52].

Cystic follicles, as opposed to growing follicles and the corpus lutea, are more prevalent in the ovaries of the PCOS group, suggesting that physiological activity in the ovaries is being inhibited in this population. There was a marked improvement in cyst reduction, and the number of Graafian follicles and corpus lutea were considerably decreased in the disease control group of mice compared to the normal group. The Graafian follicular numbers were considerably higher than the PCOS group, suggesting enhanced ovarian function. The rate of corpus luteum formation was considerably greater for the TC group. Primitive follicle counts in the ovaries of the therapy groups were, thus, considerably greater than in the PCOS group. Finally, the number of oocytes was considerably reduced in the PCOS group. The total number of oocytes produced followed a generally rising trend across all treatment groups. Polycystic ovarian syndrome is associated with anovulation due to increased androgen levels. Follicular atresia is thought to be caused by androgens binding to cell receptors and causing cell death. The oocytes in the follicles degenerate as a result of androgens, which increase the number of pyknotic granulosa cells.

## 5. Conclusions

The results of the study show that mice with DHEA-induced PCOS treated with TC preparations as nutritional supplements maintained a normal estrous cycle and gained a normal amount of weight. TC satva and oil were found to be the most effective in lowering blood sugar levels, OGTT, testosterone, LH/FSH ratio, lipid profile, insulin resistance, and ovary diameter and weight, and reducing granular changes and disease severity. Treatment of PCOS mice with TC satva and oil increased the levels of estrogen and progesterone, the number of follicles, insulin sensitivity, and FSH. Based on these results, we conclude that TC preparations as nutritional supplements may have beneficial effects in the treatment and management of PCOS-associated conditions and complications. Further studies may be required to establish the molecular mechanism of action of TC extracts for generalization and possible clinical applications.

## Data Availability

All data generated in the study are provided in the manuscript. No extra data are available.
